# Analysis of prognosis, genome, microbiome, and microbial metabolome in different sites of colorectal cancer

**DOI:** 10.1186/s12967-019-2102-1

**Published:** 2019-10-29

**Authors:** Yang Xi, Pan Yuefen, Wu Wei, Qi Quan, Zhuang Jing, Xu Jiamin, Han Shuwen

**Affiliations:** 1 Department of Oncology, Huzhou Central Hospital, Affiliated Central Hospital HuZhou University, 198 Hongqi Rd, Huzhou, 313000 Zhejiang People’s Republic of China; 2 Department of Gastroenterology, Huzhou Central Hospital, Affiliated Central Hospital HuZhou University, 198 Hongqi Rd, Huzhou, 313000 Zhejiang People’s Republic of China; 3 Graduate School of Nursing, Huzhou University, No. 1 Bachelor Road, Huzhou, 313000 Zhejiang People’s Republic of China

**Keywords:** Microbiome, Bacteria, Fungi, Metabolites, Colorectal cancer, Prognosis

## Abstract

**Background:**

The colorectum includes ascending colon, transverse colon, descending colon, sigmoid colon, and rectum. Different sites of colorectal cancer (CRC) are different in many aspects, including clinical symptoms, biological behaviour, and prognosis.

**Purpose:**

This study aimed to analyse prognosis, genes, bacteria, fungi, and microbial metabolome in different sites of CRC.

**Methods:**

The Surveillance, Epidemiology, and End Results (SEER) database and STAT were used to statistically describe and analyse the prognosis in different sites of CRC. RNA sequences of CRC from Broad Institute’s GDAC Firehose were re-annotated and reanalysed based on different sites using weighted gene co-expression network analysis (WGCNA). The Kaplan–Meier method was used to analyse the prognosis and Cytoscape was used to construct a drug-target network based on DGIdb databases. Bacterial 16S V3–V4 and fungal ITS V3–V4 ribosomal RNA genes of stool samples were sequenced. Gas chromatography/mass spectrometry (GS/MS) was performed to detect the microbial metabolites in stool samples. Bioinformatics analysis was performed to compare distinct gut microorganisms and microbial metabolites between rectal and sigmoid cancers.

**Results:**

The prognosis in CRC with different sites is significantly different. The closer to the anus predicted longer survival time. The difference between genes and co-expression pairs in CRC with different sites were constructed. The relative abundance of 112 mRNAs and 26 lncRNAs correlated with the sites of CRC were listed. Nine differentially expressed genes at different sites of CRC were correlated with prognosis. A drug-gene interaction network contained 227 drug-gene pairs were built. The relative abundance of gut bacteria and gut fungus, and the content of microbe-related metabolites were statistically different between rectal and sigmoid cancers.

**Conclusions:**

There are many differences in prognosis, genome, drug targets, gut microbiome, and microbial metabolome in different colorectal cancer sites. These findings may improve our understanding of the role of the CRC sites in personalized and precision medicine.

## Introduction

The length of the colorectum in healthy adults is about 1 m, mainly including the ascending colon, transverse colon, descending colon, sigmoid colon, and rectum depending on location [1]. Colorectal cancer (CRC) is one of the most common malignancies, and morbidity and mortality are rapidly increasing [2, 3]. According to the anatomical location, the colorectal cancer is divided into the right-sided CRC including the ascending colon and the right half of the transverse colon cancer, and the left-sided CRC including the sigmoid colon, the descending colon and the left half of the transverse colon.

The clinical features of left-sided and right-sided CRC were different in outcomes, prognoses, and clinical responses to chemotherapy [4]. Most studies showed that the prognoses of patients with left-sided CRC were better than those of patients with right-sided CRC [5–8]. Intestinal obstruction is more likely to occur on the left-sided because the right-sided colon wall is thin and easy to expand [9–11]. The more abundant blood supply in the right-sided colon results in faster tumour growth, more prone to necrosis, bleeding, and secondary infection and leads to the more common clinical characteristics including anaemia, emaciation, fever, and dyscrasia [12, 13]. From the perspective of pathological classification, the right-sided CRC has more mucinous type cancer, poorly differentiated cancer, and advanced tumour-node-metastasis stage [14]. The right-sided CRC is more frequently *BRAF* mutated, deficient in mismatch repair and microsatellite instability from the tumour molecule [6, 15, 16]. The division of left-sided and right-sided CRC also affects clinical decisions. For instance, the cetuximab (epidermal growth factor receptor inhibitor) has a better effect than bevacizumab (vascular endothelial growth factor receptor inhibitor) in left-sided CRC patients with wild-type *K*-*RAS*, *N*-*RAS* and *BRAF* genes [17].

It may be important to reiterate the pathological relevance of the CRC sites in the management and implication on future clinical and scientific research of personalized medicine. However, we forward our view that it is not detailed enough for the left–right division of CRC, which is about 1 m. It is even more unreasonable to make an early clinical prediction and select therapeutic regimen based on the left–right division. In the present study, the location of CRC was divided in more detail to analyse prognosis, genes, proteins, bacteria, fungi, and microbial metabolome.

The SEER Program, as a public database, provides numerous cancer-related data and statistics among the U.S. population [18, 19]. We attempted to clarify that the CRC at different sites has different prognosis by retrieving and analysing the sites and outcome of CRCs in the SEER database. The occurrence of CRC is the result of the combined action of genetic factors and environmental factors [20]. With the progress of molecular biology technology in recent years, it has been found that many molecular signalling pathways, such as PI3K/AKT signalling pathway, and Wnt/β-catenin signalling pathway, are involved in the pathogenesis of CRC [21–24]. The colorectum, as the storage place of faeces, has the largest number and diversity of microorganisms in the human body. These microorganisms are greatly influenced by the acquired diet, living habits, living, and working environment [25]. Micro ecological environment composed of microorganisms and microbial related metabolites is related to the occurrence and development of CRC [26–29]. Many gut microbes, such as *Fusobacteria, Streptococcus* and *Clostridium* [25, 30–32], and microbial metabolites, such as hydrolytic, reductive enzymes [33], O(6)-methyl guanine [34], short chain fatty acids and secondary bile acids, are involved in the occurrence of CRCs [26, 35, 36]. Thus, these factors, including prognosis, RNAs, bacteria, fungi, and microbial metabolites, were selected as indicators to analyse the differences at different sites of CRC in the present study.

## Materials and methods

### SEER database retrieval

SEER*Stat software (seer.cancer.gov/seerstat) was used to access the SEER data after signing a research data agreement. All patients diagnosed with colon and rectal adenocarcinoma were included. An analysis of colorectal cancer cases from 2006 to 2015, with follow-up through 2017 was conducted. The included samples were divided according to different sites, including the cecum, ascending colon, transverse colon, descending colon, sigmoid colon, and rectum. Multivariate overall survival analysis and multivariate CRC-specific survival analysis were carried out, and K–M survival analysis was used to analyse the relationship between the sites and prognosis. The alignment nomogram was constructed to describe the contribution of clinical features to prognosis. The SEER database retrieval strategy is shown in Additional file 1: Figure S1.

### Differential gene screening

Differential gene analysis strategy is shown in Additional file 2: Figure S2. Clinical data and RNA-seq (exon quantification) data were obtained from Broad Institute’s GDAC Firehose (http://gdac.broadinstitute.org). CRCs at different sites, including ascending colon, transverse colon, descending colon, sigmoid colon, and rectum, were included. The RNA-seq data were re-annotated with lncRNA and mRNA, and then screened for the differentially expressed RNA by using the EdgeR TMM normalization (Version 3.4,http://www.bioconductor.org/packages/release/bioc/html/edgeR.html) [37, 38].

Weighted gene co-expression network analysis (WGCNA) was used to analyse the co-expressed gene module. WGCNA of R package (Version 1.61, https://cran.r-project.org/web/packages/WGCNA/) [39] was used to screen for gene sets associated with colon sites. Soft-threshold (power) analysis was used to perform the Pearson correlation analysis for the expression profile and construct a weighted network. The power value was defined as the square of log(k) in the network and log(p(k)) correlation coefficient fist reached 0.95. In module mining, the minimum number of genes for each module is limited to 30, and a threshold of 0.25 is used to combine similar modules. Module eigengenes (MEs) are the main components of the gene principle component analysis in a module, and they represent the overall expression mode of this module. The phenotypic correlation modules were found by calculating the correlation between MEs and traits. Downstream analysis was performed for the modules with the most significant correlation under each phenotype (The largest correlation coefficient and p < 0.05). The coexpression network of the genes (lncRNA/mRNA) was constructed by Cytoscape software [40]. The two genes with the correlation coefficient weight more than 0.1 were included in the network.

The Database for Annotation, Visualization and Integrated Discovery (DAVID) v6.8 was used to perform gene ontology (GO), biological process (BP), and Kyoto Encyclopaedia of Genes and Genomes (KEGG) pathway analysis for modules with significant correlation among CRC sites.

The Kaplan–Meier method was used to analyse the relationship between differentially expressed genes and prognosis. The relationship between the screened differential RNA and drug targets was predicted based on drug prediction databases DGIdb (version: 3.0, http://www.dgidb.org/search_interactions) [41]. Cytoscape is used to construct a drug-target network.

### Stool samples collection

The patients with CRC admitted to the department of gastrointestinal surgery in Huzhou Central Hospital from January 2018 to December 2018 were recruited. All patients were pathologically diagnosed with adenocarcinoma and volunteered for the study. CRC patients with distant organ metastasis, complicating other gut diseases, such as ulcerative colitis and Crohn’s disease, complicating multiple primary tumours, and known primary organ failure were excluded. Approximately 30–50 g of stool samples were collected before treatment (chemotherapy, radiotherapy or surgery) and stored at − 80 °C within half an hour of sample collection. The stool samples were eliminated in the CRC patients with diarrhoea, constipation, or bloody stool, the use of oral microbial agents within 1 month, the use of a purgative or lubricant within 1 week prior to collection. The ethical approval and the informed consent form were approved by the Chinese Clinical Trial Registry (http://www.chictr.org.cn, No. ChiCTR1800018908) and Ethics Committee of Huzhou Central Hospital (No. 201601023).

### Detection of gut microorganisms

#### PCR amplification of bacterial 16S rDNA and fungal ITS rDNA

Prior to gut microorganism detection, the stored stool samples were tested for quality, including storage time, colour, weight, and stool characteristics. The E.Z.N.A.^®^ Stool DNA Kit (Omega Bio-Tek, Norcross, GA, U.S.) was used to extract total DNA. The nanodrop ND-1000 spectrophotometer (LabTech, Washington, DC, USA) with absorbances at 260 nm and 280 nm (A260/A280) was used to detect the purity of DNA. The integrity and quality of DNA were detected by electrophoresis on 2.0% (w/v) agarose gel. The PCR amplification reaction included 5 μl of DNA template, 2 μl of Nextera XT Index Primer 1 (10 μM), 2 μl of Nextera XT Index Primer 2 (10 μM), and 16 μl of ddH_2_O. The primer sequences were as follows: 16S V3–V4 rDNA: forward, CCTACGGGNGGCWGCAG; reverse, GACTACHVGGGTATCTAATCC. ITS rDNA: forward, 5′-CTTGGTCATTTAGAGGAAGTAA-3′ and reverse, 5′-GCTGCGTTCTTCATCGATGC-3′). The PCR amplification procedure was as follows, 95 °C for 3 min, 25 cycles at 95 °C for 30 s, 55 °C for 30 s, 72 °C for 45 s, and 72 °C for 5 min. Three biological replicates and three technical replicates were performed for each experiment. The 2% agarose gels, AxyPrep DNA Gel Extraction Kit (Axygen Biosciences, Union City, CA, USA) and QuantiFluor™-ST (Life Technologies, Invitrogen) were used to extract, purify and quantify the amplicons, respectively.

#### Sequencing of bacterial 16S rDNA and fungal ITS rDNA

MiSeq library was constructed as follows: the PCR products were ligated with the Y adapter. The magnetic nanoparticles were used to remove self-ligated Y adapters. The pooled DNA products constructed an Illumina Pair-End library and treated with NaOH solution. The amplicon library was sequenced using the Illumina MiSeq platform (Shanghai BIOZERON Co., Ltd.) according to standard protocols. The raw reads were deposited into the NCBI Sequence Read Archive (SRA) database.

#### Sequencing data analysis

The raw data were optimized as follows: sequences without primers were removed using Cutadapt version 1.11. The allowable error for primer matching process was 0.15. PE reads were assembled using Pandaseq, version 2.9. The allowable overlap was longer than 10 bp. The mosaic sequences longer than 300–480 bp or reads with an average quality score less than 20 were discarded. After filtering and trimming, the sequencing data was used for clustering OTUs (operational taxonomic units) and taxonomic analysis. The clean reads of 16S rDNA sequences were analysed and compared based on the Silva database (Release 119 http://www.arb-silva.de) [42]. The ITS rDNA sequences were analysed and compared based on Unite database (Release 6.0 http://unite.ut.ee/index.php) [43]. The Mothur software [44] was used to acquire the taxonomy information. The OTUs were annotated, clustered using RDP-classifier and Qiime software. The number of species in each sample was estimated by OTUs with 97% similarity. R package HCLUST (http://sekhon.berkeley.edu/stats/html/hclust.html) was used to analyse diversities and community structures.

### Microbial metabolites detection

#### Stool samples preparation

The stored stool samples were tested for quality, including storage time, colour, weight, and stool characteristics. Approximately 10 mg lyophilised stool samples were homogenised with 300 µl NaOH solution (homogenizer, BB24, Next Advance, Inc., Averill Park, NY, USA) and centrifuged at 4 °C and 16,000 rpm for 20 min (Microfuge 20R,Beckman Coulter, Inc., Indianapolis, IN, USA). The 200 µl of supernatant was transferred to an auto-sampler vial. The residue was treated with 200 µl of cold methanol. A 167 µl supernatant transferred into another auto-sampler vial after centrifugation at 4000 rpm for 20 min at 4 °C. The robotic multipurpose sample MPS2 with dual heads (Gerstel, Mulheim, Germany) was used to make the mixtures in the auto-sampler vial derivatization. The specific procedure was as follows: added 20 µl methyl chloroformate (MCF), shaken for 30 s, added another 20 µl MCF for second-time derivatization, added 400 ml CHCl_3_ and 400 ml of Na_2_CO_3_ solution (50 mmol), centrifuged at 4000 rpm for 20 min at 4 °C. The CHCl_3_ layer at the bottom was transferred to an auto-sampler vial preloaded with 25 mg anhydrous Na_2_SO_4_, shaken at 1500 rpm for 20 min at 4 °C and, then transferred to a capped empty auto-sampler vial.

#### GC-TOFMS analysis

The microbial metabolites were quantitatively detected by the Agilent 6890 N gas chromatography coupled with time-of-flight mass spectrometry (GC-TOFMS) system (Pegasus HT, Leco Corp., St. Joseph, MO, USA) in electron ionization (EI) mode. The specific parameters for GC-TOFMS analysis were as follows: Rxi-5MS capillary column was Crossbond ^®^ 5% diphenyl/95% dimethyl polysiloxane) 30 m (length) × 250 µm I.D., 0.25-µm film thickness. The helium (99.9999%) was as a carrier gas; the flow rate was 1.0 ml/min. The temperature gradient was as follows: 45 °C for 1 min, 45–260 °C (20 °C/min), 260–320 °C (40 °C/min) and 320 °C (2 min). Electron impact ionization (− 70 eV) was at a mass range of 38–550 Da. The source temperature was 220 °C. The acquisition rate was 20 spectra per second.

#### Data analysis

The stool sample and data control procedure were followed according to the criterion (ISO9001, QAIC/CN/170149, Metabo-Profile, Shanghai, China). The proprietary XploreMET software (v2.0, Metabo-Profile, Shanghai, China) was used to process the raw data from GC/TOFMS analysis. The XploreMET software can automatically process the following, including baseline correction, smoothing, peak picking, and peak signal, library searching, and area calculation. GC/MS workstation software was used to identify the differential metabolites through automatically comparing the fragment mass to charge ratio and abundance of characteristic ion fragmentation patterns. The NISI II standard muss spectral databases, the Finch databases linked to Chrom TOF software and the available reference standards in our lab was used as reference standards. The allowable similarity was more than 70%.

## Statistical analysis

The data were indicated with the mean ± standard deviation. The Student’s *t* test or SNK test was appropriately used to analyse the statistical difference in data between groups. The Chi square test was used to analyse the data count and ratio. The rank sum test was used for data that did not conform to the normal distribution. A two-tailed p < 0.05 was considered statistically significant. The SPSS Statistics version 16.0, Microsoft Excel 2007, software packages in R studio were used for calculation and plotting graphs. Special algorithms were noted in the legends of Tables and Figures.

## Results

### Prognosis in different sites of CRC

The patients diagnosed with colon and rectal adenocarcinoma from 2006 to 2015 were included. A total of 179,323 CRC patients were included after screening. The clinical characteristics of CRC in different sites are shown in Table 1. The proportion of Caucasians (79.46%) is higher than that of other races. Adenocarcinoma is dominant in histologic subtype (89.54%). Moderately differentiated CRC is dominant in histologic grade (71.40%). The CRC patients with stage 0 were only 1.11%. Multivariate CRC-specific survival analysis and multivariate overall survival analysis were used to compare the prognosis of different clinical stages in patients with different sites of colorectal cancer. The results of multivariate CRC-specific survival analysis and multivariate overall survival analysis of different clinical stages in CRC patients with different sites are shown in Tables 2 and 3, respectively. The results suggested that the ascending colon cancer has a better prognosis than other colon cancers.

**Table 1:** Clinical characteristics of CRC in different sites in the SEER database

	Cecum	Ascending colon	Transverse colon	Descending colon	Sigmoid colon	Rectum	Total
Median age (min–max)	71 (15–105)	71 (15–108)	70 (13–102)	65 (16-101)	64 (11–106)	61 (14–103)	67 (11–108)
Gender (%)							
Male	9.93	8.81	4.25	3.01	13.81	10.76	50.57
Female	12.62	10.48	4.54	2.62	11.95	7.22	49.43
Race (%)							
White	18.29	15.38	6.97	4.11	20.04	14.67	79.46
Black	2.92	2.47	1.13	0.89	2.67	1.43	11.51
Asian	1.09	1.19	0.56	0.55	2.61	1.57	7.57
Other	0.25	0.25	0.13	0.08	0.45	0.31	1.46
Stage at presentation (%)							
Stage 0	0.28	0.25	0.08	0.06	0.29	0.15	1.11
Stage I	4.69	4.25	1.72	1.03	6.02	4.18	21.90
Stage II	6.73	6.69	3.25	1.84	7.11	4.71	30.32
Stage III	7.23	5.77	2.53	1.87	8.39	7.27	33.06
Stage IV	3.61	2.33	1.20	0.83	3.95	1.67	13.60
Histologic subtype (%)							
Adenocarcinoma	19.23	16.54	7.73	5.13	24.21	16.70	89.54
Mucinous adenocarcinoma	2.59	2.20	0.88	0.42	1.30	0.97	8.37
Other	0.73	0.55	0.18	0.08	0.25	0.31	2.10
Histologic grade (%)							
Well differentiated	1.92	1.66	0.74	0.50	2.34	1.38	8.54
Moderately differentiated	14.89	12.73	6.01	4.17	19.75	13.85	71.40
Poorly differentiated	4.88	4.20	1.77	0.85	3.25	2.47	17.43
Undifferentiated	0.85	0.69	0.27	0.12	0.43	0.27	2.63
Adequate lymph node examination (more than 12 nodes) (%)							
Yes	18.68	16.59	6.67	4.20	18.47	11.95	76.56
No	3.81	2.66	2.10	1.43	7.23	5.93	23.17
Missing data	0.06	0.04	0.02	0.01	0.06	0.09	0.27
Type of surgery (%)							
Local excision	0.02	0.03	0.01	0.02	0.11	0.26	0.45
Partial colectomy	3.59	2.79	3.54	1.91	20.36	12.41	44.60
Hemicolectomy	18.39	16.05	4.90	3.48	4.24	0.72	47.77
Total colectomy	0.29	0.22	0.19	0.15	0.51	3.69	5.04
Other colectomy	0.24	0.18	0.14	0.08	0.52	0.79	1.94
No surgery	0.02	0.02	0.01	0.01	0.03	0.12	0.19
Chemotherapy (%)							
Yes	7.33	5.52	2.74	2.14	9.93	12.41	40.07
No/unknown	15.21	13.77	6.05	3.49	15.83	5.57	59.93
Radiation therapy (%)							
Yes	0.32	0.15	0.06	0.07	0.81	11.38	12.79
No/unknown	22.23	19.14	8.73	5.56	24.95	6.60	87.21

SEER*Stat software (seer.cancer.gov/seerstat) was used to access the SEER data after signing research data agreement. All patients diagnosed with colon and rectal adenocarcinoma from 2006 to 2015 were included. A total of 179,323 CRC patients were included after screening

**Table 2:** Multivariate CRC-specific survival analysis of different clinical stages in CRC patients with different sites

	Cecum	Ascending colon HR (95% CI)	Transverse colon HR (95% CI)	Descending colon HR (95% CI)	Sigmoid colon HR (95% CI)	Rectum HR (95% CI)
Stage at presentation						
Stage 0	1.00	0.64 (0.48–0.85)*	1.18 (0.8–1.74)	1.25 (0.91–1.74)	0.98 (0.69–1.4)	1.14 (0.71–1.84)
Stage I	1.00	0.58 (0.54–0.61)**	0.86 (0.79–0.93)**	1.07 (1–1.15)*	1.03 (0.95–1.1)	1.05 (0.96–1.16)
Stage II	1.00	0.57 (0.54–0.6)**	0.78 (0.73–0.84)**	1 (0.95–1.06)	0.94 (0.89–0.99)*	0.91 (0.85–0.98)*
Stage III	1.00	0.55 (0.52–0.58)**	0.81 (0.75–0.87)**	1.1 (1.03–1.18)*	0.98 (0.91–1.05)	0.87 (0.8–0.95)*
Stage IV	1.00	0.58 (0.5–0.66)**	0.88 (0.75–1.04)	1.11 (0.96–1.28)	1.07 (0.92–1.24)	0.94 (0.78–1.13)
All stages	1.00	0.56 (0.54–0.57)**	0.82 (0.78–0.85)**	1.07 (1.04–1.11)**	0.98 (0.95–1.02)	0.94 (0.89–0.98)*

** p < 0.001, *p < 0.05; *CI* confidence interval, *HR* hazard ratio. The classification criteria of stages refer to the AJCC/UICC TNM staging system. The cecum cancer group was set to 1 as the control group

**Table 3:** Multivariate overall survival analysis of different clinical stages in CRC patients with different sites

	Cecum	Ascending colon HR (95% CI)	Transverse colon HR (95% CI)	Descending colon HR (95% CI)	Sigmoid colon HR (95% CI)	Rectum HR (95% CI)
Stage at presentation						
Stage 0	1.00	0.77 (0.6–0.99)*	1.37 (0.96–1.96)	1.36 (1.01–1.83)*	1 (0.72–1.39)	1.07 (0.68–1.68)
Stage I	1.00	0.72 (0.68–0.75)**	1.02 (0.95–1.09)	1.15 (1.08–1.21)**	1.07 (1.01–1.14)*	1.07 (0.98–1.16)
Stage II	1.00	0.81 (0.78–0.84)**	0.92 (0.88–0.96)**	0.96 (0.93–1)	1.01 (0.97–1.05)	0.94 (0.89–0.99)*
Stage III	1.00	0.63 (0.61–0.65)**	1 (0.96–1.04)	1.12 (1.08–1.16)**	1.05 (1.01–1.09)*	0.93 (0.88–0.97)*
Stage IV	1.00	0.6 (0.58–0.62)**	0.94 (0.89–0.98)*	1.07 (1.03–1.11)*	0.95 (0.91–0.99)*	0.93 (0.89–0.98)*
All stages	1.00	0.7 (0.69–0.71)**	0.95 (0.93–0.97)**	0.97 (0.95–0.99)*	1.04 (1.02–1.07)**	0.96 (0.93–0.98)*

** p < 0.001, *p < 0.05; *CI* confidence interval, *HR* hazard ratio. The classification criteria of stages refer to the AJCC/UICC TNM staging system. The cecum cancer group was set to 1 as the control group

Right-sided (RS) CRC includes cancers located in the cecum, ascending colon, and transverse colon, and left-sided (LS) CRC includes those located in the descending colon, sigmoid colon, and rectum. Prognosis of CRC was analysed based on the regrouping of left and right colorectal cancer. The clinical characteristics of the left and right CRC in the SEER database are shown in Table 4. The median age of RS CRC is older than that of LS CRC. The results of multivariate CRC-specific survival analysis and multivariate overall survival analysis of different clinical stages in left-sided and right-sided CRC are shown in Tables 5 and 6, respectively. The results suggested that the left-sided CRC has a better prognosis than the right-sided CRC.

**Table 4:** Clinical characteristics of left-sided and right-sided CRC in the SEER database

	RS	LS	Total
Median age (min–max)	71 (13–108)	63 (11–106)	67 (11–108)
Gender			
Male	22.99	27.58	50.57
Female	27.64	21.79	49.43
Race (%)			
White	40.65	38.82	79.47
Black	6.52	4.99	11.51
Asian	2.84	4.73	7.57
Other	0.61	0.85	1.46
Stage at presentation (%)			
Stage 0	0.61	0.50	1.11
Stage I	10.67	11.23	21.90
Stage II	16.67	13.66	30.32
Stage III	15.53	17.53	33.06
Stage IV	7.14	6.46	13.60
Histologic subtype (%)			
Adenocarcinoma	43.49	46.04	89.54
Mucinous adenocarcinoma	5.67	2.69	8.37
Other	1.45	0.64	2.10
Histologic grade (%)			
Well differentiated	4.33	4.21	8.54
Moderately differentiated	33.63	37.77	71.40
Poorly differentiated	10.85	6.58	17.43
Undifferentiated	1.81	0.82	2.63
Adequate lymph node examination (more than 12 nodes) (%)			
Yes	41.94	34.62	76.56
No	8.57	14.60	23.17
Missing data	0.11	0.16	0.27
Type of surgery (%)			
Local excision	0.06	0.39	0.45
Partial colectomy	9.93	34.68	44.60
Hemicolectomy	39.34	8.43	47.77
Total colectomy	0.70	4.34	5.04
Other colectomy	0.55	1.38	1.94
No surgery	0.04	0.15	0.19
Chemotherapy (%)			
Yes	15.59	24.48	40.07
No/unknown	35.03	24.90	59.93
Radiation therapy (%)			
Yes	0.53	12.26	12.79
No/unknown	50.09	37.12	87.21

All patients diagnosed with colon and rectal adenocarcinoma from 2006 to 2015 in the SEER database were included. Right-sided (RS) CRC includes cancers located in the cecum, ascending colon, and transverse colon, and left-sided (LS) CRC includes those located in the descending colon, sigmoid colon, and rectum. A total of 179,323 CRC patients were included after screening

**Table 5:** Multivariate CRC-specific survival analysis of different clinical stages in left-sided and right-sided CRC

	RS	LS HR (95% CI)
Stage at presentation		
Stage 0	1.00	0.72 (0.6–0.86)**
Stage I	1.00	0.71 (0.68–0.74)**
Stage II	1.00	0.74 (0.71–0.76)**
Stage III	1.00	0.69 (0.67–0.72)**
Stage IV	1.00	0.72 (0.66–0.78)**
All stages	1.00	0.71 (0.69–0.72)**

** p < 0.001, *p < 0.05; *CI* confidence interval, *HR* hazard ratio. The classification criteria of stages refer to the AJCC/UICC TNM staging system. Right-sided (RS) CRC includes cancers located in the cecum, ascending colon, and transverse colon, and left-sided (LS) CRC include those located in the descending colon, sigmoid colon, and rectum. The LS group was set to 1 as the control group

**Table 6:** Multivariate overall survival analysis of different clinical stages in left-sided and right-sided CRC

	RS	LS HR (95% CI)
Stage at presentation		
Stage 0	1.00	0.79 (0.68–0.93)*
Stage I	1.00	0.79 (0.77–0.82)**
Stage II	1.00	0.89 (0.87–0.91)**
Stage III	1.00	0.74 (0.73–0.76)**
Stage IV	1.00	0.74 (0.73–0.76)**
All Stages	1.00	0.81 (0.8–0.82)**

** p < 0.001, *p < 0.05; *CI* confidence interval, *HR* hazard ratio. The classification criteria of stages refer to the AJCC/UICC TNM staging system. Right-sided (RS) CRC includes cancers located in the cecum, ascending colon, and transverse colon, and left-sided (LS) CRC includes those located in the descending colon, sigmoid colon, and rectum. The LS group was set to 1 as the control group

**Fig. 1:**
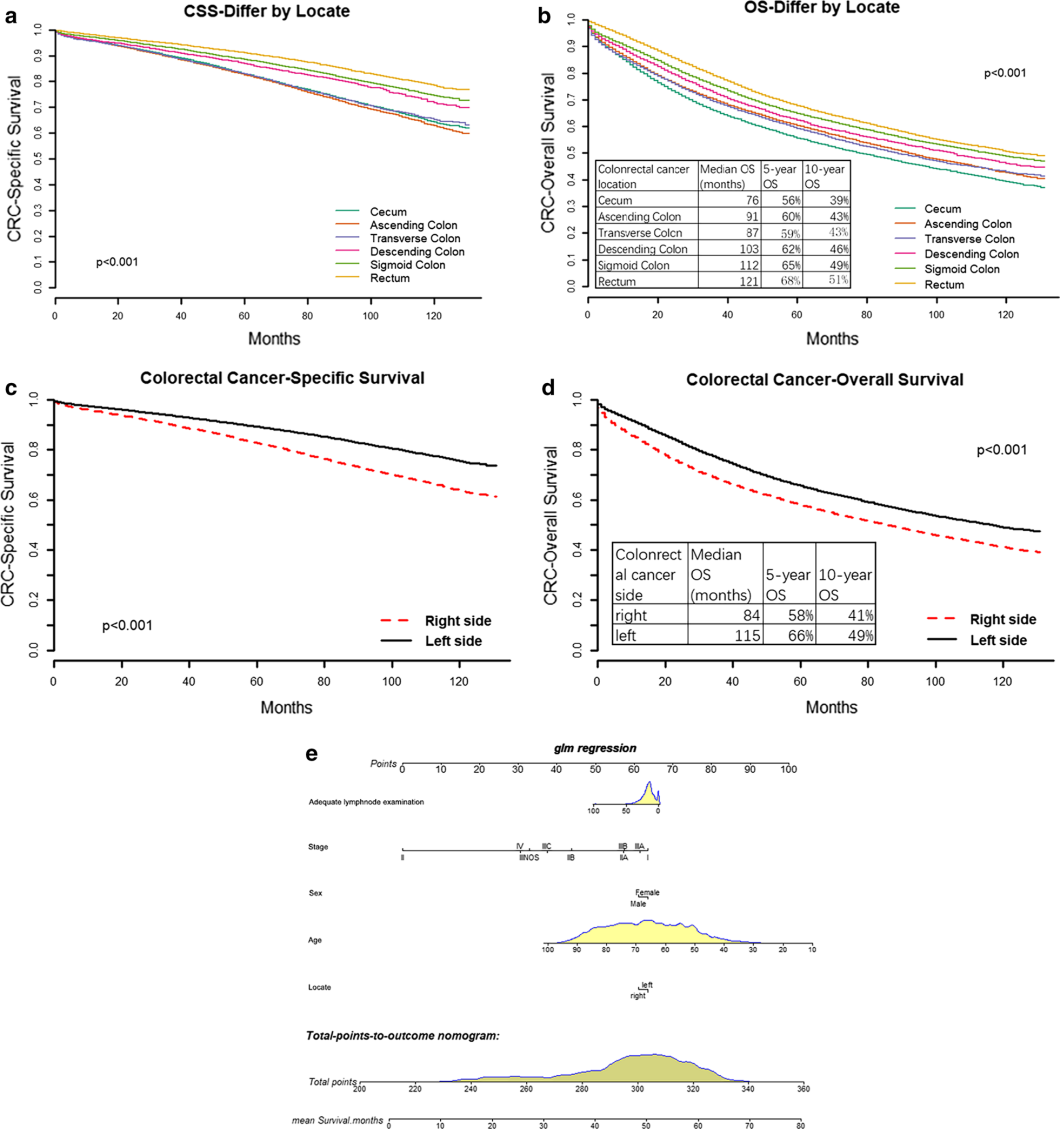
Prognosis in different sites of CRC. CRC can be divided into right-sided CRC and left-sided CRC according to the pathogenic site. Right-sided CRC includes cancers located in the cecum, ascending colon, and transverse colon, and left-sided CRC includes those located in the descending colon, sigmoid colon, and rectum. The clinical data from the SEER database was used to analyze the prognosis in different sites of CRC. **a**, **b** The CRC specific survival rate and overall survival rate varied among the different sites, respectively. **c**, **d** The CRC specific survival rate and overall survival rate between the right-sided CRC and left-sided CRC, respectively. **e** A nomogram model to be used for predicting the prognosis of CRC via some prognostic factors, including adequate node examination, stages, gender, age, and sites

The CRC specific survival rate and overall survival rate varied among the different site’s areas and are shown in Fig. 1a, b. The results suggested the 10-year survival time of patients with different sites of CRC was cecum, transverse colon, ascending colon, descending colon, sigmoid colon, and rectum, in order.

The CRC specific survival rate and overall survival rate varied between the left-sided and right-sided CRC (Fig. 1c, d). The results suggested that the 10-year survival time of patients with right-sided CRC was longer than that of patients with left-sided CRC. Moreover, as shown in Fig. 1e, based on prognostic factors including adequate node examination (more than 12 nodes), stages, gender, age and sites, a nomogram model was constructed and used for predicting mean survival months.

### Genome in different sites of CRC

A total of 217 clinical samples with RNA-seq data from Broad Institute’s GDAC Firehose were included. The samples were divided into five groups including ascending colon (54 cases), descending colon (14 cases), rectum (41 cases), sigmoid colon (84 cases) and transverse colon (24 cases). Table 7 shows the clinical characteristics of CRC. The gene with annotation information of “protein_coding” was as mRNA, the annotation information for “antisense”, “sense_intronic”, “lncRNA”, “sense_overlapping”, “processed_transcript”, “3 prime_overlapping_ncRNA”, “non_coding” as lncRNA genes. A total of 1868 lncRNAs and 7848 mRNAs were obtained.

**Table 7:** Clinical characteristics of CRC in different sites from Broad Institute’s GDAC Firehose

	Rectum group	Colon group	p-value
Cases, n	41	176	–
Males, n	24	93	0.510
Age (years)	44.88 ± 14.26	43.90 ± 14.55	0.697
Vital status	8	42	0.551
Days to death (days)	599.88 ± 468.76	853.74 ± 806.69	0.395
NA	8	134	–
Days to last follow-up (days)	876.48 ± 691.48	995.50 ± 952.63	0.501
NA	8	42	–
Pathologic stage			
Stage I	4	22	0.698
Stage II	15	74	
Stage III	15	48	
StageVI	6	24	
NA	1	8	–
Pathology T stage			
T1	2	4	0.757
T2	4	23	
T3	30	125	
T4	5	24	
Pathology N stage			
N0	19	104	0.062
N1	12	50	
N2	9	22	
NX	1	0	
Pathology M stage			
M0	31	120	0.279
M1	6	24	
MX	3	31	
NA	1	1	–

A total of 217 clinical samples with RNA-seq data from Broad Institute’s GDAC Firehose were included. The tables compiled the clinical characteristics of CRC in different sites from Broad Institute’s GDAC Firehose

**Fig. 2:**
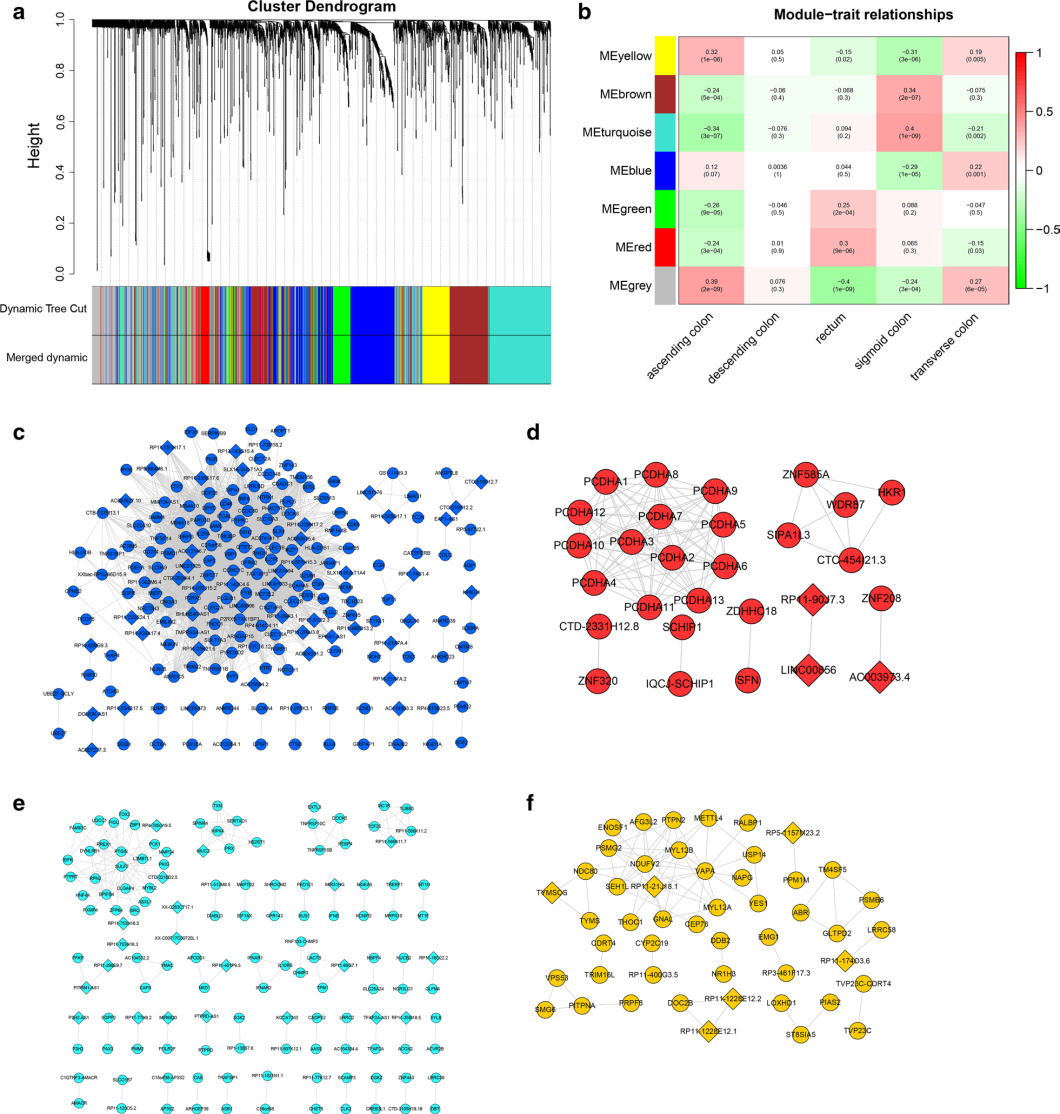
The co-expression network of different RNAs in different sites of CRC. A total of 217 clinical samples with RNA-seq data from Broad Institute’s GDAC Firehose were analysed. **a** The different modules in the gene co-expression network based on WGCNA. Each colour represents a module. **b** The correlation analysis between the modules and the sites of CRC. **c**–**f** The co-expression network with regard to transverse colon cancer, rectum cancer, sigmoid cancer and ascending colon cancer, respectively. The circle and the diamond represent mRNA and lncRNA, respectively

Differential gene analysis was performed on lncRNA and mRNA levels in the five groups, and a total of 421 differential lncRNAs and 1770 differential mRNAs were obtained. Figure S3 A-B shows the heatmap of the differentially expressed mRNA and lncRNA. The expression levels of mRNA and lncRNA were combined into one expression profile for WGCNA analysis. According to the method described in “Materials and methods” section, the power value was defined as the square of log(k) in the network and log(p(k)) correlation coefficient fist reached 0.95 (β = 6, scale free *R*^*2*^= 0.98). The key parameters including frequency of k, check scale-free topology scale, scale independence and mean connectivity are shown in Additional file 3: Figure S3 c–e. As shown in Fig. 2a, different modules in the gene co-expression network were constructed based on WGCNA. Each colour represents a module. According to the module mining method and the set threshold, the brown, turquoise, yellow, blue, green, and red contains 405, 518, 173, 465, 162 and 123 genes, respectively. The grey contains genes that cannot be classified as any module.

The correlation analysis between the modules and the sites of CRC was performed to explore the significant modules related to the sites of CRC. According to the standard set in the method, the module-trait relationship is shown in Fig. 2b. The yellow module, red module, turquoise module, and the blue module were applied to analyse the downstream genes for ascending colon cancer, rectum cancer, sigmoid colon cancer, and transverse colon cancer, respectively. No significant correlation with descending colon cancer was found. According to the threshold set in the method, the co-expression network of the four modules was constructed (Fig. 2c–f). The blue module associated with the transverse colon cancer contains 201 genes and 3230 co-expression pairs (Fig. 2c). The red module associated with rectum cancer contains 28 genes and 91 co-expression pairs (Fig. 2d). The turquoise module associated with sigmoid cancer contains 132 genes and 147 co-expression pairs (Fig. 2e). The yellow module associated with ascending colon cancer contains 49 genes and 74 co-expression pairs (Fig. 2f). The Gene Ontology biological process (GO, BP) and KEGG pathway enrichment analysis were performed for all genes contained in the four modules. The results are shown in Additional file 4: Figure S4.

**Fig. 3:**
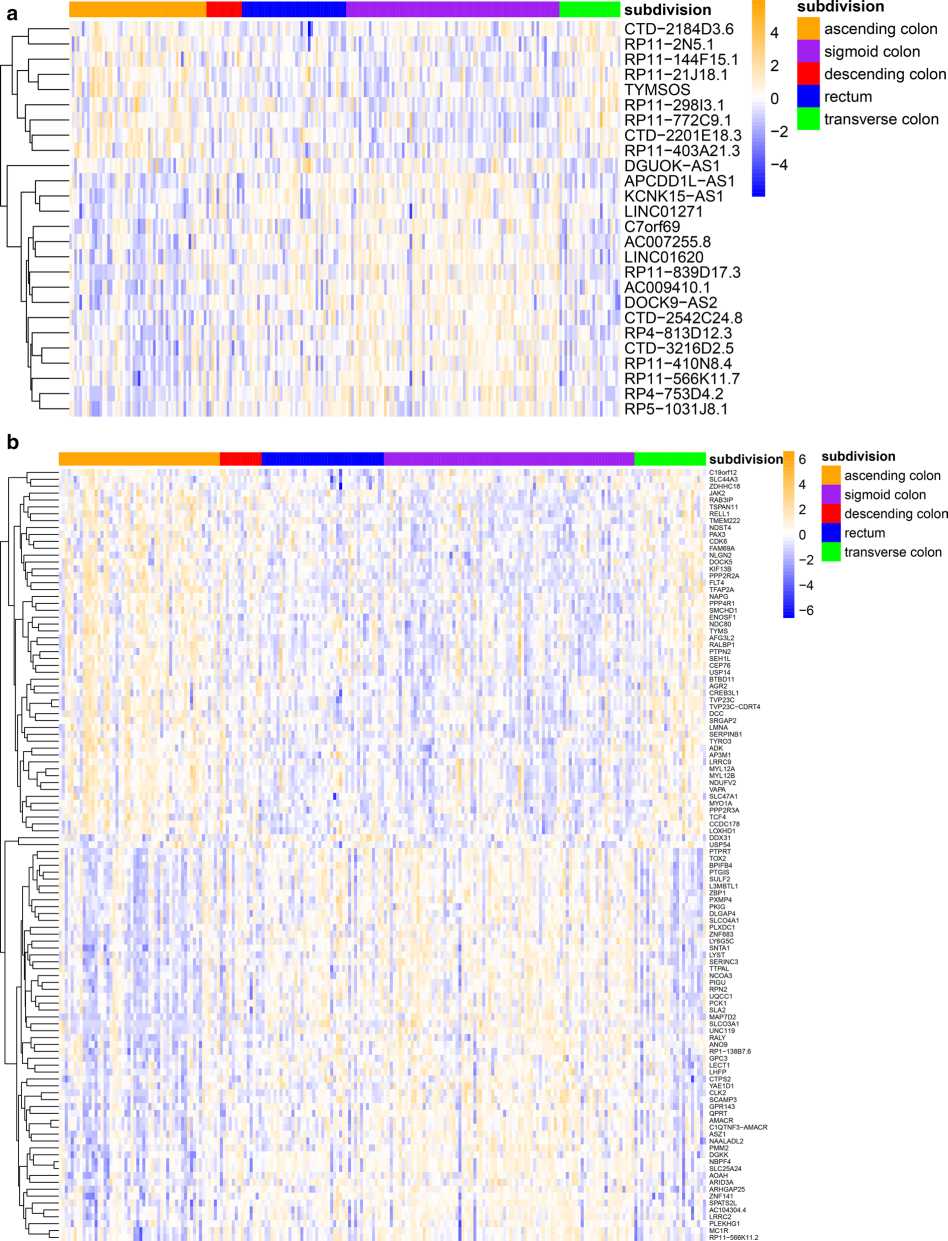
Differential RNA expression in different sites of CRC. **a** The differential expression of 26 lncRNAs in different sites of CRC. **b** The differential expression of 112 mRNAs in different sites of CRC (Chi square test, p value < 0.05)

Firstly, the Pearson correlation coefficients between all genes and the modules were calculated, and the significance test was carried out according to the set threshold value (correlation coefficient > 0.6 and p < 0.05). The results showed that the number of genes significantly correlated with the yellow module, turquoise module, blue module, and red module was 80, 132, 148, and 25, respectively. Secondly, the Pearson correlation coefficients among different sites of CRC were calculated, and the significance test was carried out according to the set threshold value (correlation coefficient > 0.2 and p < 0.05). The results showed that the number of genes significantly correlated with ascending colon cancer, sigmoid colon cancer, transverse colon cancer, and rectum cancer was 460, 733, 140 and 249, respectively. The intersection of the genes above two parts was taken, 56 yellow module genes, 106 turquoise module genes, 8 blue module genes and 7 red module genes which were found to be significantly correlated with ascending colon cancer, sigmoid colon cancer, transverse colon cancer, and rectum cancer, respectively. A total of 158 genes were identified, 19 of which were significantly associated with both the ascending and sigmoid colon. The samples were divided into high and low expression groups according to the median expression values of these 158 genes. The Chi square test was used to compare that in different sites of CRC. Finally, 138 genes including 112 mRNA and 26 lncRNA were significantly correlated with the sites of CRC. The heatmap in Fig. 3a, b represents the expression of the 26 lncRNA and 112 mRNA. The results showed that the gene expression patterns of ascending colon, descending colon and transverse colon were similar, and the gene expression patterns of sigmoid colon and rectum were also similar.

**Fig. 4:**
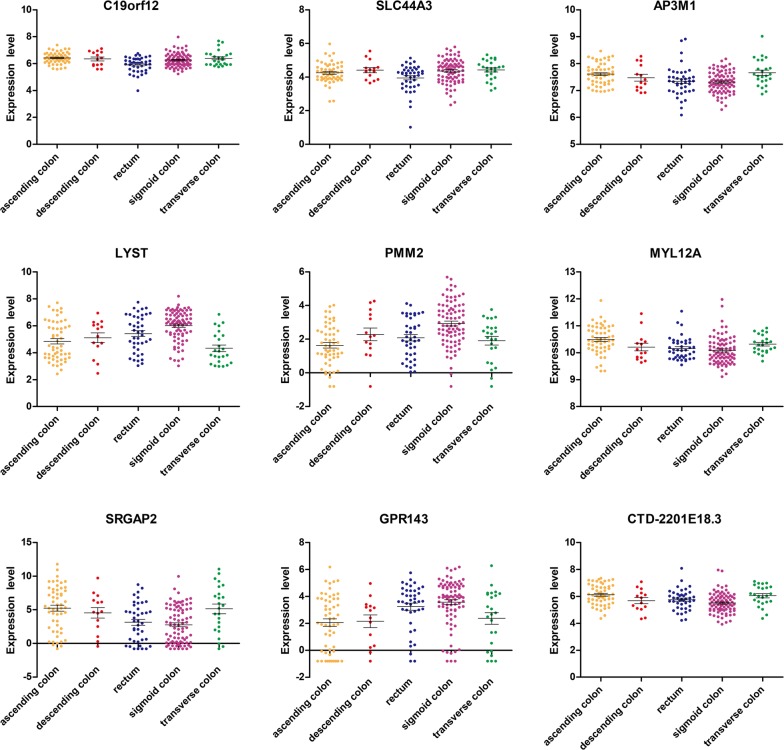
Differentially expressed genes affect prognosis in different sites of CRC. The log-rank test and K-M survival analysis were used to analyze the relationship between the prognosis and the differential expression of genes in different sites of CRC. The results showed nine genes, including *C19orf12*, *SLC44A3*, *AP3M1*, *LYST*, *PMM2*, *MYL12A*, *SRGAP2*, *GPR143,* and *CTD*-*2201E18.3* were related to the prognosis of CRC (p value < 0.05). The figure shows the gene expression distribution of these 9 genes

**Fig. 5:**
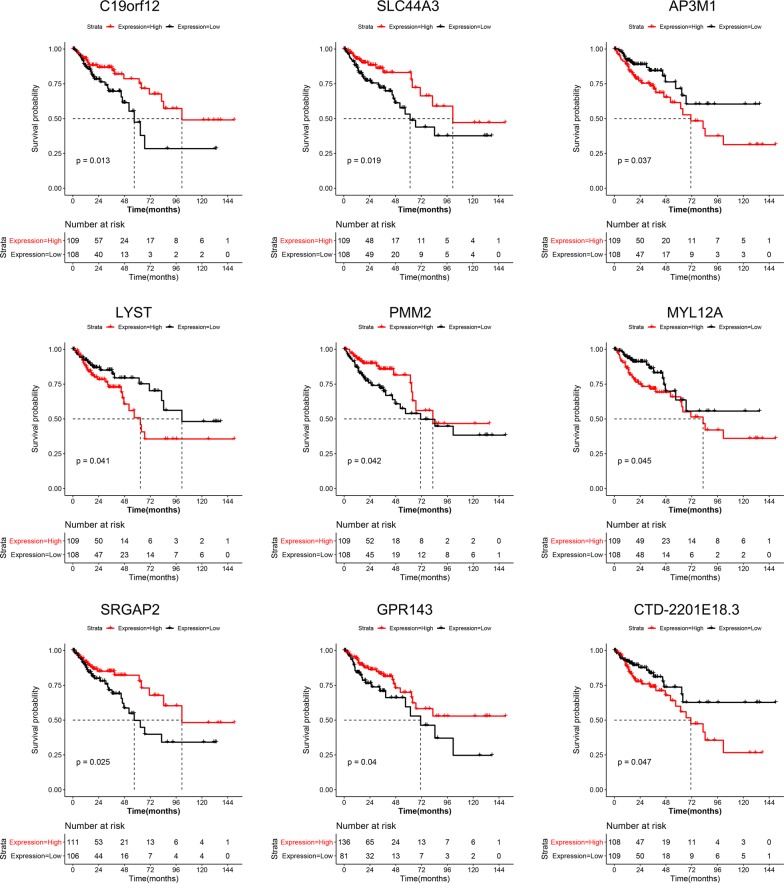
Differential genes that affect prognosis in different sites of CRC. The log-rank test and K-M survival analysis were used to analyze the relationship between the prognosis and the differential genes in different sites of CRC. The results showed nine genes, including *C19orf12*, *SLC44A3*, *AP3M1*, *LYST*, *PMM2*, *MYL12A*, *SRGAP2*, *GPR143*, and *CTD*-*2201E18.3* were related to the prognosis of CRC (p-value < 0.05). The Figure shows the survival curves of these 9 genes

K–M survival analysis was used to analyse the relationship between the above mentioned 138 genes and the prognosis. The log-rank test showed nine genes, including *C19orf12*, *SLC44A3*, *AP3M1*, *LYST*, *PMM2*, *MYL12A*, *SRGAP2*, *GPR143*, and *CTD*-*2201E18.3* had statistical significance (p < 0.05). The gene expression distribution and survival curves of these 9 genes in different sites of CRC are shown in Figs. 4 and 5, respectively.

**Fig. 6:**
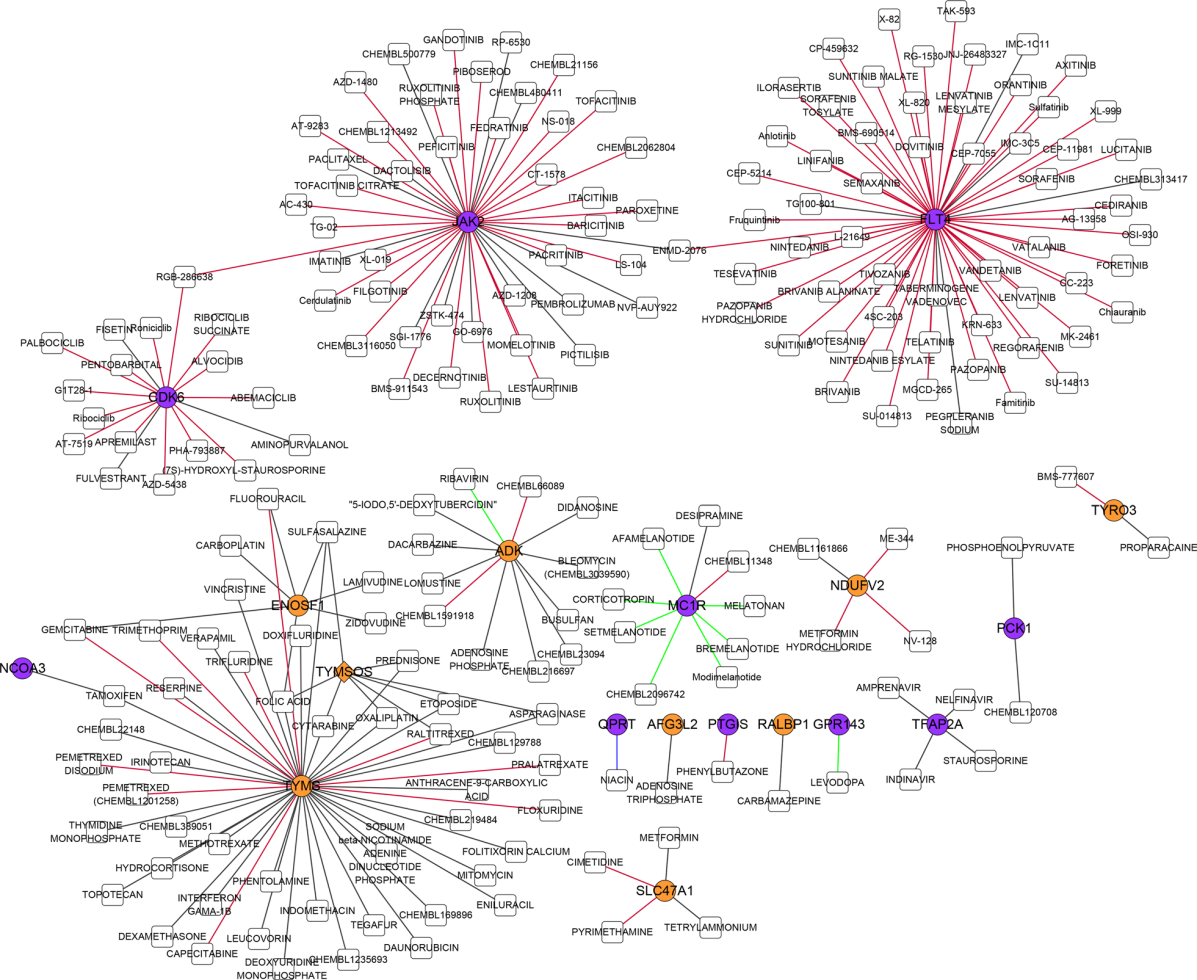
The network of a drug-gene interaction. The drug prediction databases DGIdb was used to predict drugs that interact with the differentially expressed genes in different sites of CRC. The Figure shows the network of drug-gene interaction. The yellow circles and purple circles represent the genes significantly associated with the ascending colon and the sigmoid colon, respectively. The white squares indicate predicted drugs. The red lines, green lines, and black lines represent the antagonistic effects, promoting effects, and unknown effects, respectively

The drug prediction database DGIdb was used to predict drugs that interact with these 138 genes. The network of drug-gene interaction is shown in Fig. 6, which indicates that there were 227 drug-gene pairs containing 212 drugs and 19 genes.

### Gut microorganisms in rectal and sigmoid cancers

A total of 28 CRC patients including 15 cases of rectal cancer and 13 cases of sigmoid cancer in Huzhou Central Hospital from January 2018 to August 2018 were recruited for the microbiology and microbial metabolites study after screening and identification. The clinical characteristics of these case are shown in Table 8.

**Table 8:** Clinical characteristics of CRC involving the microbiological study

	Rectum group	Sigmoid colon group	p-value
Cases, n	15	13	–
Age (years)	59.53 ± 12.14	68.15 ± 9.75	0.051
Males, n	9	7	0.743
BMI (kg/m^2^)	22.96 ± 3.03	22.90 ± 2.91	0.956
Known hypertension, n	4	6	0.283
Known diabetes, n	2	0	0.172
Long-term Smoking history, n	3	3	0.843
Long-term drinking history, n	3	2	0.750
Red blood cells (10^12^/L)	4.38 ± 0.67	4.16 ± 0.51	0.336
Haemoglobin (g/L)	132.20 ± 21.55	124.69 ± 19.79	0.349
white blood cells (10^9^/L)	5.53 ± 1.38	4.88 ± 1.38	0.225
Blood platelet (10^9^/L)	228.73 ± 77.88	192.54 ± 52.56	0.168
Haematocrit	0.40 ± 0.06	0.40 ± 0.05	0.321
Mean red blood cell haemoglobin content	30.20 ± 1.90	29.65 ± 3.35	0.594
Mean corpuscular-haemoglobin concentration	331.40 ± 12.02	329.08 ± 13.85	0.639
Mean red blood cell volume	91.12 ± 5.27	89.90 ± 7.50	0.619
Red blood cell distribution width	12.78 ± 0.64	13.35 ± 2.12	0.332
Alanine transaminase (U/L)	15.97 ± 8.23	15.05 ± 5.40	0.732
Glutamic oxaloacetic transaminase (U/L)	18.93 ± 2.88	18.39 ± 2.18	0.585
Total protein (g/L)	66.19 ± 7.24	65.92 ± 5.39	0.910
Albumin (g/L)	37.78 ± 4.35	37.40 ± 3.86	0.810
Total bilirubin (g/L)	13.57 ± 7.79	13.00 ± 5.70	0.828
Direct bilirubin (g/L)	5.01 ± 2.62	4.60 ± 1.59	0.631
Creatinine (μmol/L)	70.61 ± 15.91	72.52 ± 15.60	0.753
Urea nitrogen (mmol/L)	5.56 ± 2.11	4.39 ± 1.43	0.101
Glucose (mmol/L)	5.13 ± 0.98	4.93 ± 0.60	0.536
CA125	10.31 ± 2.93	10.96 ± 3.17	0.579
CA153	8.09 ± 4.71	6.88 ± 3.26	0.444
CA724	2.21 ± 1.56	2.36 ± 2.20	0.843
CA199	9.12 ± 9.28	38.19 ± 50.70	0.038
CEA	19.62 ± 37.26	10.56 ± 15.23	0.421
Cytokeratin 19 fragment	1.61 ± 0.60	2.03 ± 0.84	0.137
Neuron specific enolase	11.69 ± 3.07	9.85 ± 2.09	0.102
Squamous cell carcinoma associated antigen	0.83 ± 0.59	0.73 ± 0.27	0.598
Alpha fetoprotein	3.20 ± 1.93	2.37 ± 0.74	0.154
Ki-67 (%)	61.33 ± 11.87	65.38 ± 13.91	0.413
TMN stage			
Stage II	13	8	0.126
Stage III	2	5	

A total of 28 CRC patients including 15 cases of rectal cancer and 13 cases of sigmoid cancer in Huzhou Central Hospital from January 2018 to August 2018 were recruited in the microbiology and microbial metabolites study after screening and identification. Smoking and drinking history over the course of 1 year were recruited. The TMN stages are according to AJCC. Patient personal information, serological indicators, and Ki-67 indexes were collected from the hospital’s HIS system after the consent of the patients

**Fig. 7:**
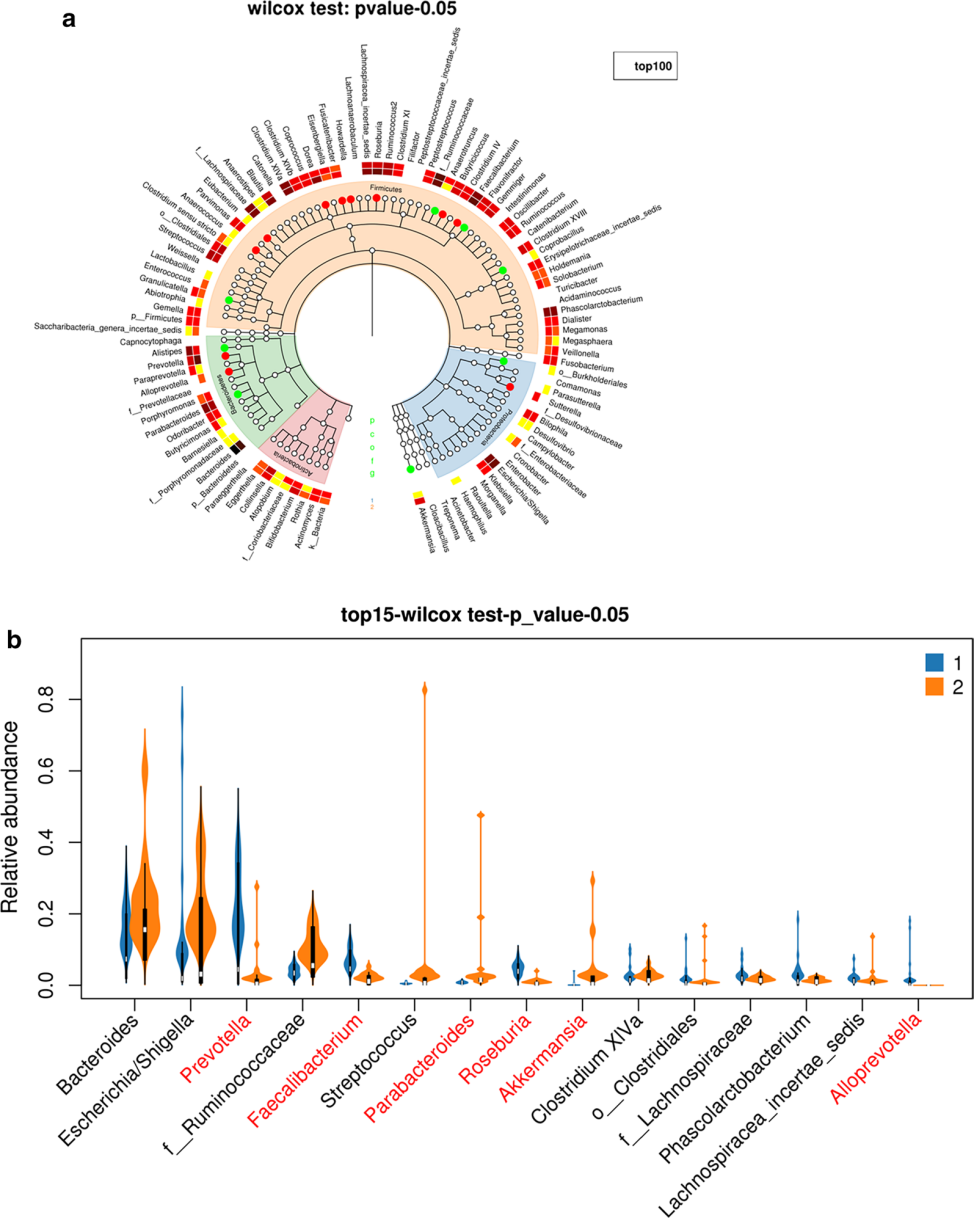
Different bacterial community structure between rectal cancer and sigmoid cancer. Rectal cancer and sigmoid cancer was marked “1” group and “2” group, respectively. **a** The taxonomic tree heatmap, which displayed the relative abundance ratio of gut bacteria in the top 100 at different taxonomic levels. The innermost layer shows the taxonomic tree. The circle from the inside to the outside represents different taxon levels from the phylum to a genus. The white circles represent no statistical difference, the red circles represent the higher abundance of species in rectal cancer group, and the cyan circles represent the higher abundance of species in sigmoid cancer group (Kruskal test, p-value < 0.05). The outermost layer is bacterial annotation at the genus level. **b** The violin plot which displayed the relative abundance of gut bacteria in rectal cancer and sigmoid cancer in the top 15 at the genus levels. The red letters represent statistically different in the abundance of bacteria

The alpha diversity analysis was carried out to compare the diversity of gut microorganism in rectal cancer and that in sigmoid cancer. The Chao1 index, Shannon index and Simpson index are indicators of microbial diversity. Additional file 5: Figure S5 A–C shows the Chao1 curves, Shannon curves, and Simpson curves, respectively. Higher Chao1 and Shannon value indicate higher community diversity. Lower Simpson value indicates a higher community diversity. The box chart on the right in Additional file 5: Figure S5 indicates no statistical difference of these alpha diversity indexes about gut bacteria between rectal cancer (marked “1” group) and sigmoid cancer (marked “2” group) (Kruskal test, p > 0.05). The analysis of bacterial community structure is shown in Fig. 7. The taxonomic tree heatmap in Fig. 7a shows the relative abundance ratio of gut bacteria in the top 100 at different taxonomic levels. The taxonomic tree heatmap indicates that the relative abundance of gut bacteria including *Prevotella, Eubacterium, Dorea, Fusicatenibacter, Howardella, Butyricicoccus, Anaerococcus, Alloprevotella, Faecalibacterium, Roseburia,* and *Sutterella* in rectal cancer were more than that in sigmoid cancer at genus level, and the relative abundance of gut bacteria including *Granulicatella, Burkholderiales, Flavonifractor, Coprobacillus, Parabacteroides, Anaerotruncus, Akkermansia, Allisonella,* and *Alistipes* in rectal cancer were less than that in sigmoid cancer (Wilcox test, p < 0.05). The violin plot in Fig. 7b displayed the relative abundance of gut bacteria in rectal cancer and sigmoid cancer in the top 15 at the genus levels. The results showed that there were statistically significant differences in these bacteria, including *Prevotella, Faecalibacterium, Parabacteroides, Roseburia, Akkermansia,* and *Alloprevotella* between the two groups (Wilcox test, p < 0.05).

**Fig. 8:**
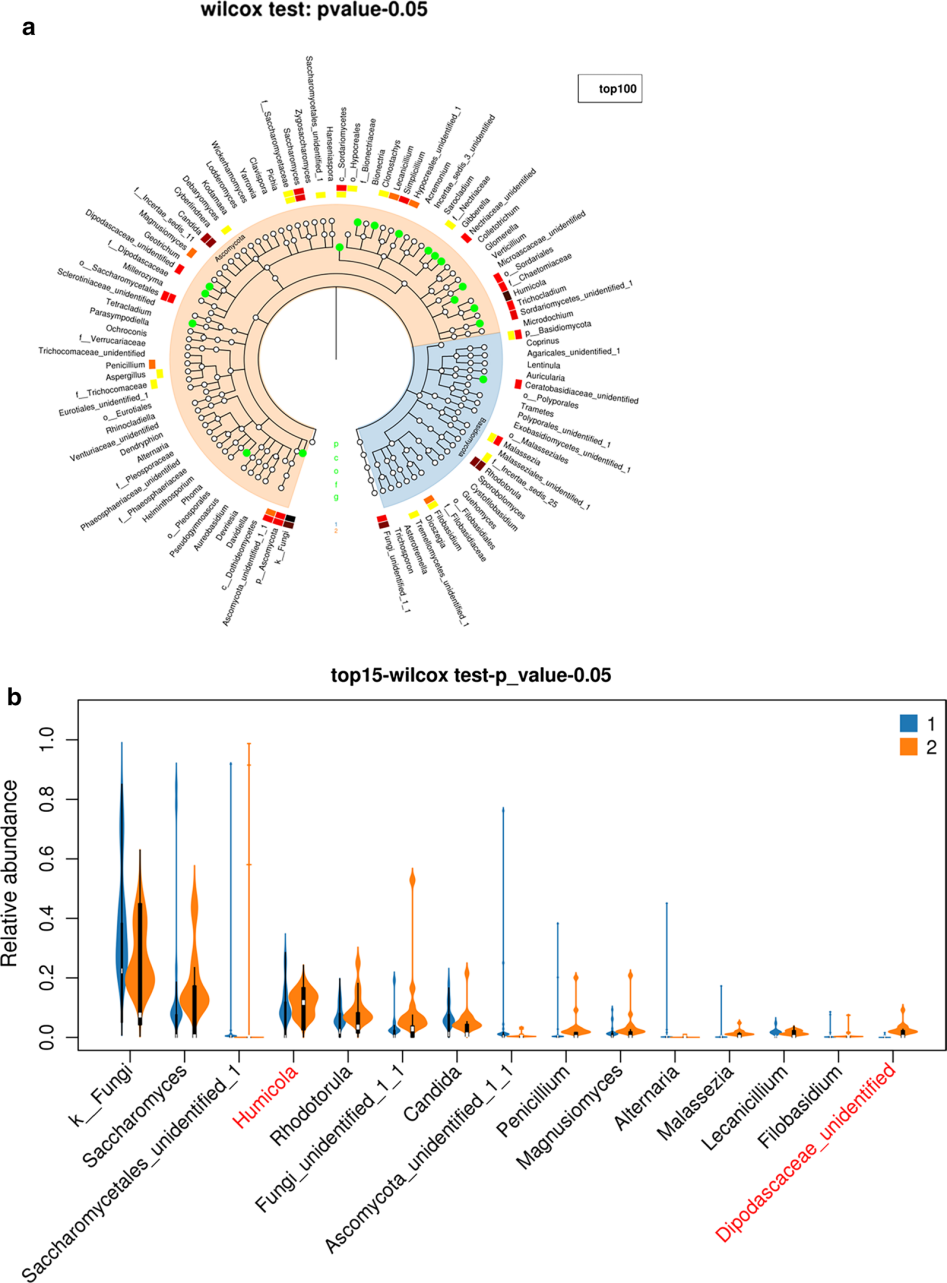
Different fungal community structure between rectal cancer and sigmoid cancer. Rectal cancer and sigmoid cancer was marked “1” group and “2” group, respectively. **a** The taxonomic tree heatmap, which displayed the relative abundance ratio of gut fungus in the top 100 at different taxonomic levels. The innermost layer shows the taxonomic tree. The circle from the inside to the outside represents different taxon levels from the phylum to the genus. The white circles represent no statistical difference, and the cyan circles represent the higher abundance of species in the sigmoid cancer group (Kruskal test, p-value < 0.05). The outermost layer is fungal annotation at the genus level. **b** The violin plot, which displayed the relative abundance of gut fungus in rectal cancer and sigmoid cancer in the top 15 at the genus levels. The red letters represent statistically different in the abundance of fungus

The Chao1 curves, Shannon curves, and Simpson curves of gut fungus in rectal cancer and sigmoid cancer are shown in Additional file 6: Figure S6 A–C. The results showed that there was no statistical difference of these alpha diversity indexes of gut fungus between rectal cancer (marked “1” group) and sigmoid cancer (marked “2” group)(Kruskal test, p > 0.05). The taxonomic tree heatmap in Fig. 8a shows the relative abundance of gut fungus including *Humicola, Dipodascaceae, Nectriaceae, o_Pleosporales, f_Nectriaceae, Tetracladium, Clonostachys, f_Dipodascaceae, o_Sordariales, Microascaceae, Ceratobasidiaceae, c_Sordariomycetes, Gibberella, o_Hypocreales* and *Sordariomycetes* in rectal cancer were less than that in sigmoid cancer at the genus level. The violin plot in Fig. 8b displays that there were statistically significant differences in these two fungi, including *Humicola* and *Dipodascaceae* in rectal cancer and sigmoid cancer in top 15 at genus levels.

### Microbial metabolites in rectal and sigmoid cancers

**Fig. 9:**
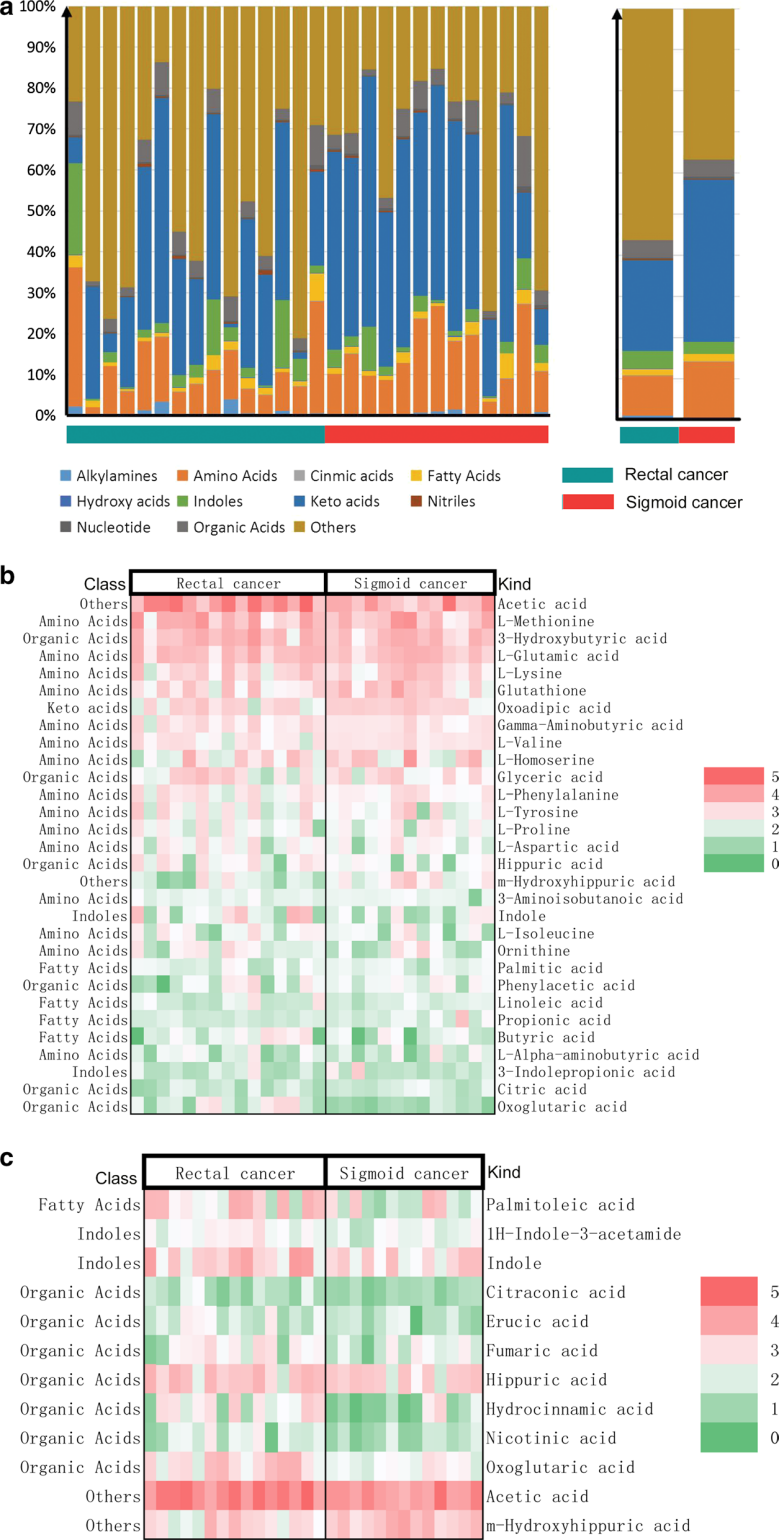
Different microbial metabolites between rectal cancer and sigmoid cancer. **a** The left shows the composition and proportion of microbial metabolites in rectal and sigmoid cancers at the class level. The **a** on the right shows the concentration of microbial metabolites in the same site combined into a group. The number of nanogram metabolites per milligram of faeces was analysed. The logarithm of metabolite content (ng/mg) was taken to build the heatmap for better display. **b** The content of the top 30 microbial metabolites in rectal and sigmoid cancers. **c** The content of microbial metabolites with statistical differences between the two groups (Kruskal test, p-value < 0.05)

Figure 9 shows that the composition and proportion of microbial metabolites in rectal and sigmoid cancers at the class level. The concentration of microbial metabolites in the same site was combined into a group. The result suggests that the proportion of fatty acids from patients with rectal cancer was higher than that from patients with sigmoid cancers, while the proportion of amino acids and hydroxy acids from patients with rectal cancer was lower than that from patients with sigmoid cancer. Figure 9b shows the content of the top 30 microbial metabolites in rectal and sigmoid cancers. The results showed that the top 5 microbial metabolites were, in order, acetic acid, l-methionine, 3-hydroxybutyric acid, l-glutamic acid, and l-lysine. Figure 9c and Table 9 show that the content of microbial metabolites with statistical differences between the two groups. The results showed that the microbial metabolites with statistical differences between rectal and sigmoid cancers including *palmitoleic acid,* ^*1*^*H*-*indole*-*3*-*acetamide, indole, citraconic acid, erucic acid, fumaric acid, hippuric acid, hydrocinnamic acid, nicotinic acid, oxoglutaric acid, acetic acid,* and *m*-*hydroxyhippuric acid.*

**Table 9:** Differences in microbial metabolites between rectal cancer and sigmoid cancer

	Rectal cancer	Sigmoid cancer	p-value
Cases, n	15	13	–
Palmitoleic acid (ng/mg)	1033.65 ± 1074.68	315.39 ± 529.27	0.038
1H-Indole-3-acetamide (ng/mg)	96.36 ± 69.24	46.84 ± 22.90	0.021
Indole (ng/mg)	1876.80 ± 2381.81	494.15 ± 473.88	0.050
Citraconic acid (ng/mg)	21.10 ± 19.11	9.42 ± 3.63	0.040
Erucic acid (ng/mg)	53.32 ± 52.14	19.54 ± 12.96	0.032
Fumaric acid (ng/mg)	141.11 ± 129.74	51.75 ± 47.20	0.027
Hippuric acid (ng/mg)	1182.46 ± 833.71	641.37 ± 369.39	0.040
Hydrocinnamic acid (ng/mg)	217.46 ± 243.41	57.57 ± 135.79	0.046
Nicotinic acid (ng/mg)	47.59 ± 50.16	16.86 ± 12.71	0.041
Oxoglutaric acid (ng/mg)	795.68 ± 897.00	90.56 ± 81.18	0.009
Acetic acid (ng/mg)	26,194.35 ± 21,949.09	11,351.32 ± 13,454.89	0.044
m-Hydroxyhippuric acid (ng/mg)	500.98 ± 711.27	1413.65 ± 1354.92	0.031

The number of nanogram metabolites per milligram of faeces were analysed by GC-TOFMS analysis. The table showed the content of microbial metabolites with statistical differences between the two groups (Kruskal test, p-value < 0.05)

## Discussion

Analysis of the effect of different pathogenic sites on the prognosis of CRC suggested the RS CRC has a better prognosis than the LS CRC, consistent with several studies [7]. The present study further explored the correlation between different pathogenic sites, including the cecum, transverse colon, ascending colon, descending colon, sigmoid colon and rectum, and prognosis of CRC. The results suggested that the closer to the anus predicted longer survival time, which will be favourable to the accurate judgment of clinical prognosis of diseases.

The differential expression of mRNA and lncRNA in different pathogenic sites of CRC were analysed based on RNA-seq data from Broad Institute’s GDAC Firehose. The analysis of these differential gene expressions provides further research direction for the pathogenesis and prognosis of different CRC sites. The expression levels of some genes, for instance, *K*-*RAS*, *N*-*RAS*, *BRAF, p53*, *Ki*-*67*, and MSI (microsatellite instability) classification have been used to predict the prognosis of CRC [45–48]. The search for more gene targets will contribute to accurately determine the treatment regimen decision and prognosis evaluation of CRC. Nine genes with different prognosis were initially identified based on the classification of pathogenic sites of CRC.

Small molecule targeted drugs for the treatment of CRC recommended by the NCCN guidelines mainly includes anti-EGFR such as cetuximab and anti-VEGF such as bevacizumab and panitumumab [49]. The introduction of more targeted drugs into clinical applications and the patients with advanced disease states seeking more targeted drugs in clinical trial phase put higher demands on the prediction of drug targets and the selection of targeted drugs. The network of drug-gene interaction annotated in this study will provide a reference for gene target detection and drug selection based on the classification of pathogenic sites of CRC.

The colorectum has the largest microbial community in the body. The gut microecological environment may be the incentive of somatic genetic and epigenetic changes in CRC. Gut microorganisms cannot be ignored in the study of CRC. The results showed that the abundance of many gut bacteria and gut fungus and the content of many microbial related metabolites between rectal and sigmoid cancers are statistically different between rectal and sigmoid cancers. On the one hand, the results explained the differences between microorganisms and microbial related metabolites in different pathogenic sites of CRC. On the other hand, the intervention, such as faecal transplantation, oral administration of microorganisms or microbial metabolites and dietary choices may provide alternative and auxiliary treatments for CRC patients. The results may provide a new idea for the microbiological treatment decision.

The era of big data provides tremendous data support for medical research. The integration of various databases for the diagnosis and treatment of diseases will effectively promote the development of human health. The present study was based on the SEER database to analyze the influence of the incidence site on the prognosis of CRC. RNA-sequence data from Broad Institute’s GDAC Firehose provide the data support for the genomic analysis of different CRC sites. Drug prediction database DGIdb provides accurate guidance for drug target screening of differential genes. The microbiological analysis also depends on some databases, including Silva database and Unite database. Based on the application of big data, the collision of epidemiology, genomics, microbiome, and metabonomics may lead to a new interdisciplinary study to establish a basis for individualized prevention and treatments for CRC.

There are some shortcomings in this study. The SEER database which is based on the North American population, cannot be the representative of the patients from other races and ethnicities. The results of data analysis based on RNA database still need further clinical validation. A database of correlation between microbiome and cancer has not yet been established in the low incidence of right colorectal cancer and most cases in the late stage. The sample size of the right CRC without organ metastasis was insufficient. We only included the cases of rectal and sigmoid cancers for microbiome and microbial metabolomics studies.

The following directions are put forward for future development. (i) At the Big data application level, greater data sharing will promote the development of diagnosis and treatment of colorectal cancer. Advances in applied mathematics will drive the establishment of more advanced mathematical models. It will further promote the development of precision medicine. (ii) At the basic research level, these aspects need to be further developed, such as the molecular mechanism of genes and proteins on the occurrence of CRC, the influence of microorganisms and metabolites on DNA and RNA, the relationship between microorganisms and metabolites and the identification of fungal species. (iii) In clinical application, stratification based on pathogenic sites will be taken into account in the subsequent diagnosis and treatment of CRC.

## Conclusions

In the present study, we show first the clinical data analysis from the SEER database, which indicates that the prognosis in CRC with different sites is significantly different. The general trend is that the closer to the anus predicted longer survival time. Second, the difference between genes and co-expression pairs in CRC with different sites were constructed through the analysis of RNA-seq from Broad Institute’s GDAC Firehose. The expression differences of 112 mRNA and 26 lncRNA correlated with the sites of CRC were listed. The nine differentially expressed genes correlated with prognosis were further analysed. Third, a network of drug-gene interaction was built based on the different genes at different sites using DGIdb databases. Fourth, the diversity and microbial community structure differences of gut bacteria and fungus were compared between the rectal and sigmoid cancers. Fifth, the plot and difference of gut microbial related metabolites between the rectal and sigmoid cancers were displayed. We emphasize that there are many differences with regard to prognosis, genome, drug targets, gut microbiome, and microbial metabolome among different colorectal cancer sites. Our findings, therefore, might be useful in understanding the role of the CRC sites in personalized and precision medicine.

## Supplementary information

**Additional file 1: Figure S1.** SEER database retrieval strategy. After signing a research data agreement, all patients diagnosed with colon and rectal adenocarcinoma from 2006 to 2015, with follow-up through 2017 were included.

**Additional file 2: Figure S2.** Differential gene screening and analysis strategy. The clinical data and RNA-seq (exon quantification) from Broad Institute’s GDAC Firehose (http://gdac.broadinstitute.org/) were obtained. CRC cases were divided into ascending colon, transverse colon, descending colon, sigmoid colon, and rectum. The relationship between the screened differential RNA and drug targets was predicted based on drug prediction databases DGIdb.

**Additional file 3: Figure S3.** The key parameters of differential gene screening. Differential expression of gene analysis was performed on lncRNA and mRNA expression levels in the five groups including ascending colon, transverse colon, descending colon, sigmoid colon, and rectum. A total of 421 differential lncRNAs and 1770 differential mRNAs were finally obtained. Panel A and panel B show the heatmap which described the differential mRNA and lncRNA, respectively. The differential mRNA and lncRNA were combined into one expression profile for WGCNA analysis. The power value was defined as the square of log(k) in the network and log(p(k)) correlation coefficient fist reached 0.95 (β = 6,scale free *R*^*2*^= 0.98). Soft-threshold (power) analysis was used to perform the Pearson correlation analysis for the expression profile and construct a weighted network. Panels C, D, and E show the key parameters including frequency of k, check scale-free topology scale, scale independence, and mean connectivity.

**Additional file 4: Figure S4.** Functional enrichment analysis of differentially expressed genes in different sites of CRC. The GO, BP and KEGG pathway enrichment analyses were performed for differentially expressed genes in different sites of CRC. The blue module, red module, turquoise module, and yellow module at the horizontal axis represent the transverse colon cancer, rectum cancer, sigmoid cancer, and ascending colon cancer, respectively. The vertical axis represents the GO, BP pathway and the KEGG pathway. The beginning of *hsa* and GO represents the KEGG pathway and GO, BP, respectively. The bubble size represents the number of genes enriched, and the colour ranges from blue to red represents the size of the p-value.

**Additional file 5: Figure S5.** Alpha diversity analysis of gut bacteria between rectal cancer and sigmoid cancer. Rectal cancer and sigmoid cancer was marked “1” group and “2” group, respectively. The Chao1 index, Shannon index and Simpson index are indicators of microbial diversity. Panels A, B, and C show the Chao1 curves, Shannon curves, and Simpson curves, respectively. Higher Chao1 and Shannon value indicate higher community diversity. Lower Simpson value indicates higher community diversity. The box chart on the right describes the average level and variation degree between the two groups (Kruskal test, p > 0.05).

**Additional file 6: Figure S6.** Alpha diversity analysis of gut fungus between rectal cancer and sigmoid cancer. Rectal cancer and sigmoid cancer was marked “1” group and “2” group, respectively. The Chao1 index, Shannon index, and Simpson index are indicators of microbial diversity. Panels A, B, and C show the Chao1 curves, Shannon curves, and Simpson curves, respectively. The box chart on the right describes the average level and variation degree between the two groups (Kruskal test, p > 0.05).

## Data Availability

The datasets generated during the current study are not publicly available but de-identified and anonymized information is potentially available on reasonable request.

## Abbreviations

CRC: colorectal cancer
SEER: Surveillance, Epidemiology, and End Results
WGCNA: weighted gene co-expression network analysis
GS/MS: gas chromatography/mass spectrometry
MEs: module eigengenes
DAVID: Database for Annotation, Visualization and Integrated Discovery
GO: gene ontology
BP: biological process
KEGG: Kyoto Encyclopaedia of Genes and Genomes
PCR: polymerase chain reaction
SRA: Sequence Read Archive
OTU: Operational Taxonomic Unit
TOFMS: time-of-flight mass spectrometry
MCF: methyl chloroformate
GC-TOFMS: gas chromatography coupled to time-of-flight mass spectrometry
EI: electron ionization
RS: right-sided
LS: left-sided
MSI: microsatellite instability
