# Supplementary figures and images for: Analysis of prognosis, genome, microbiome, and microbial metabolome in different sites of colorectal cancer
Source: J Transl Med. 2019 Oct 29;17:353. doi: 10.1186/s12967-019-2102-1 (PMC6819376; doi:10.1186/s12967-019-2102-1)

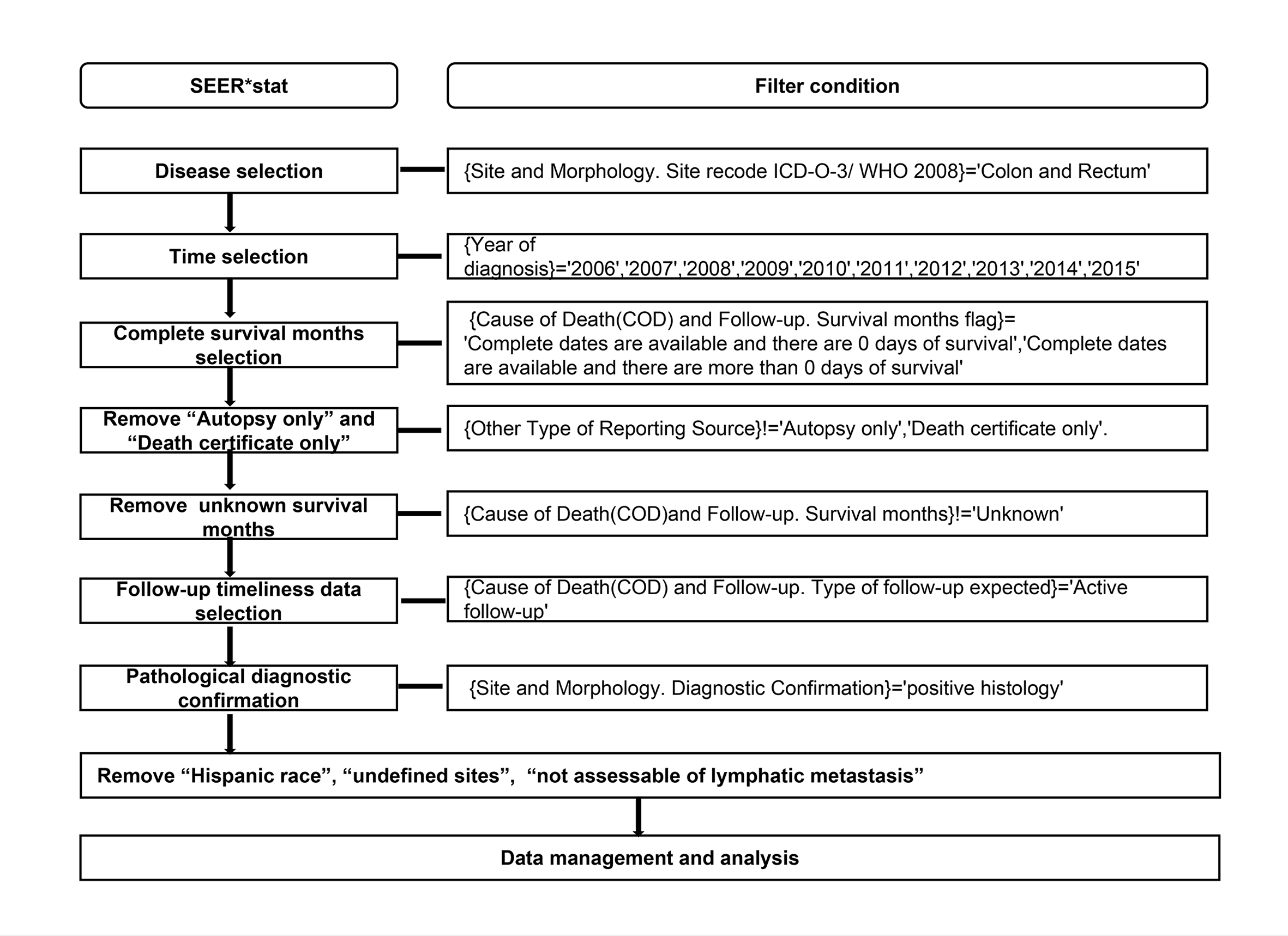

Supplement: Supplementary file 1 — Additional file 1: Figure S1. SEER database retrieval strategy. After signing a research data agreement, all patients diagnosed with colon and rectal adenocarcinoma from 2006 to 2015, with follow-up through 2017 were included. [file 12967_2019_2102_MOESM1_ESM.tif]

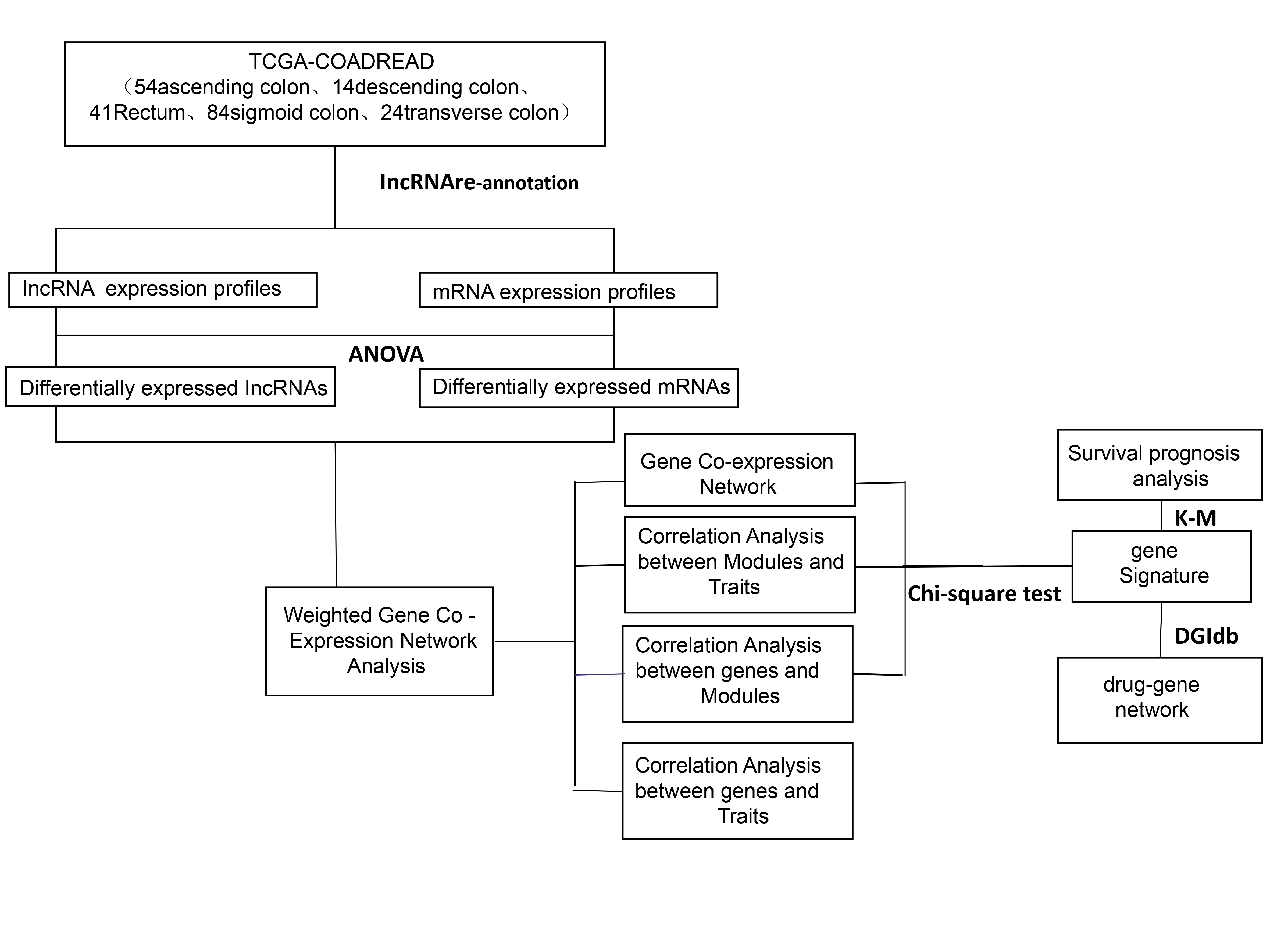

Supplement: Supplementary file 2 — Additional file 2: Figure S2. Differential gene screening and analysis strategy. The clinical data and RNA-seq (exon quantification) from Broad Institute’s GDAC Firehose (http://gdac.broadinstitute.org/) were obtained. CRC cases were divided into ascending colon, transverse colon, descending colon, sigmoid colon, and rectum. The relationship between the screened differential RNA and drug targets was predicted based on drug prediction databases DGIdb. [file 12967_2019_2102_MOESM2_ESM.tif]

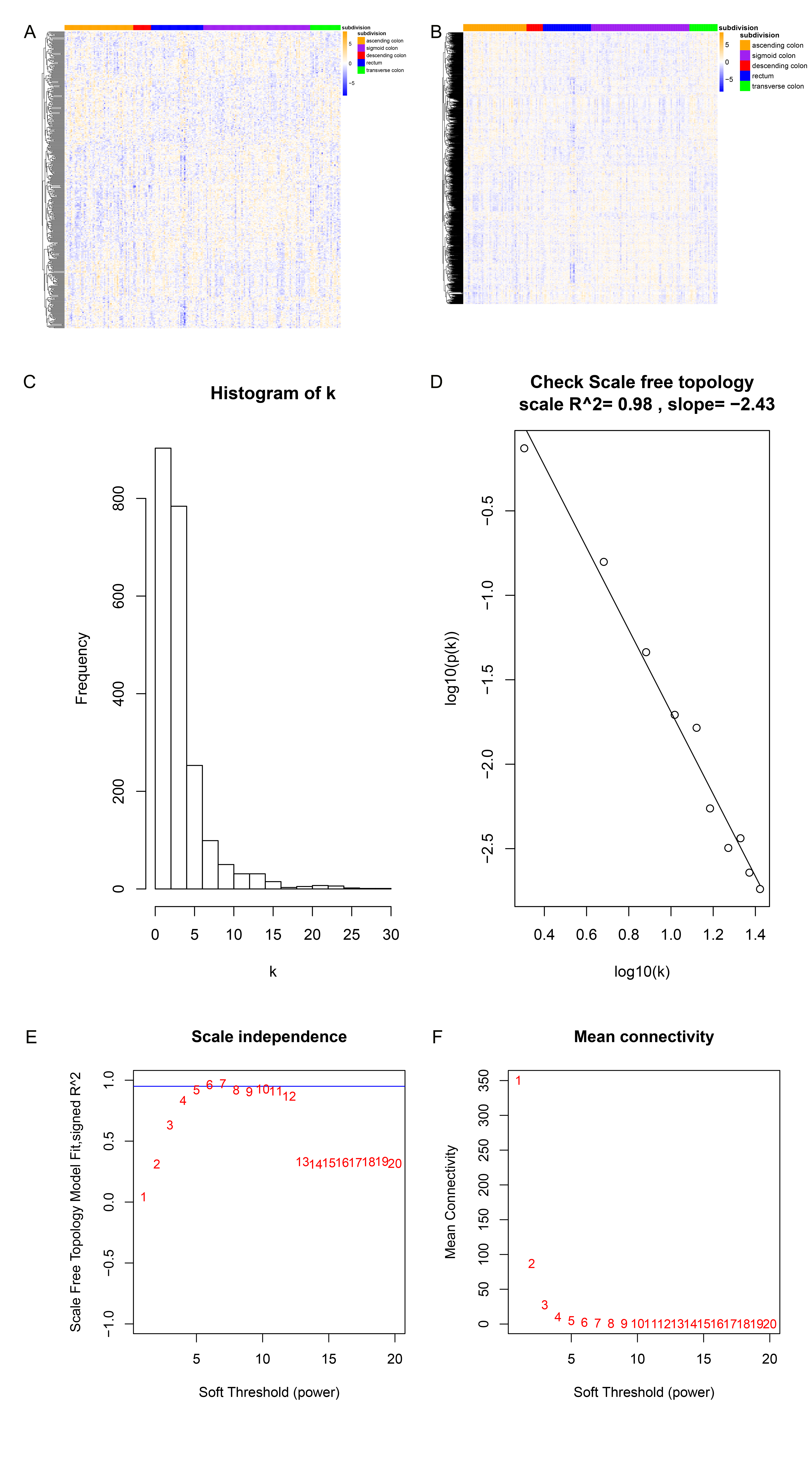

Supplement: Supplementary file 3 — Additional file 3: Figure S3. The key parameters of differential gene screening. Differential expression of gene analysis was performed on lncRNA and mRNA expression levels in the five groups including ascending colon, transverse colon, descending colon, sigmoid colon, and rectum. A total of 421 differential lncRNAs and 1770 differential mRNAs were finally obtained. Panel A and panel B show the heatmap which described the differential mRNA and lncRNA, respectively. The differential mRNA and lncRNA were combined into one expression profile for WGCNA analysis. The power value was defined as the square of log(k) in the network and log(p(k)) correlation coefficient fist reached 0.95 (β = 6,scale free R2= 0.98). Soft-threshold (power) analysis was used to perform the Pearson correlation analysis for the expression profile and construct a weighted network. Panels C, D, and E show the key parameters including frequency of k, check scale-free topology scale, scale independence, and mean connectivity. [file 12967_2019_2102_MOESM3_ESM.tif]

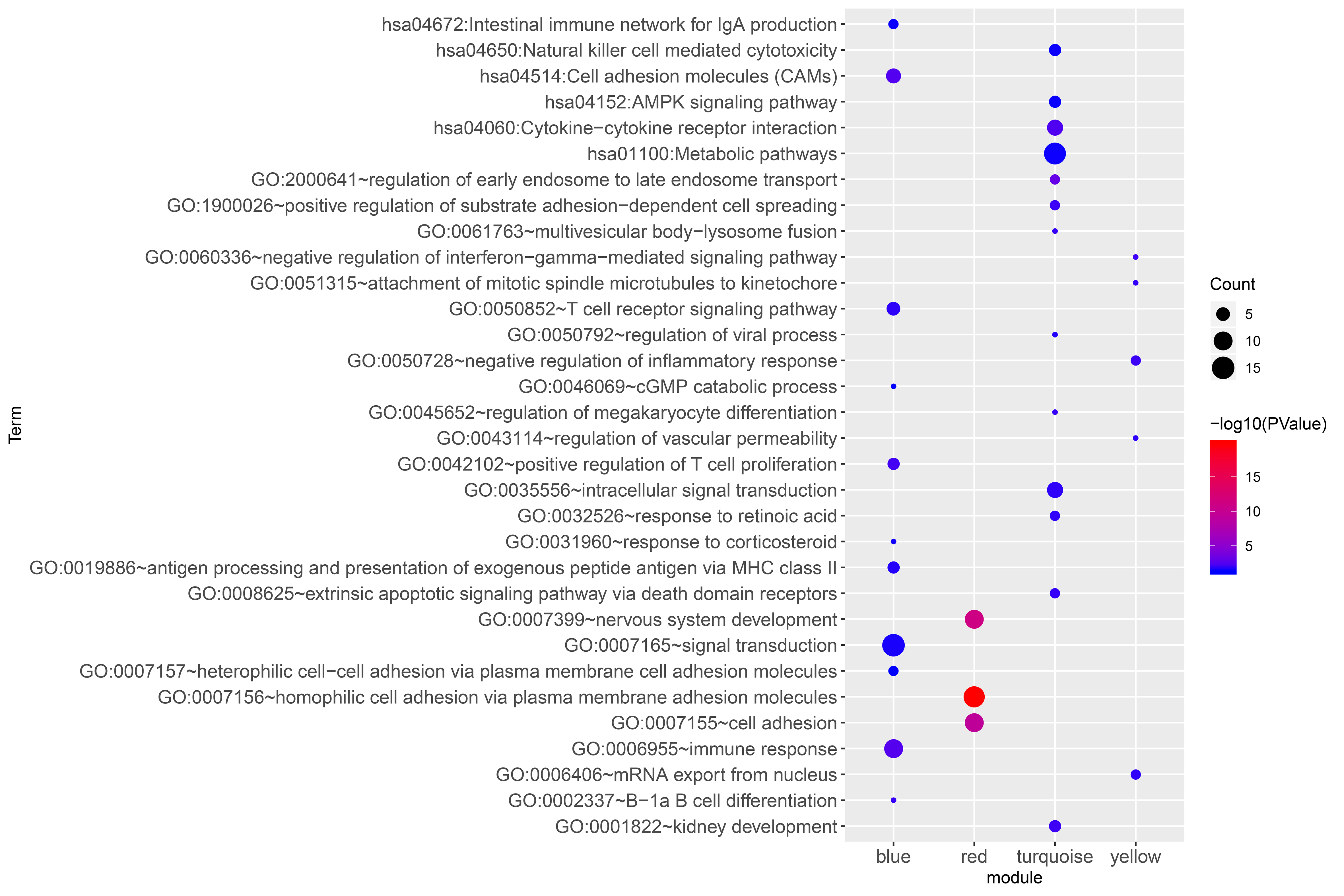

Supplement: Supplementary file 4 — Additional file 4: Figure S4. Functional enrichment analysis of differentially expressed genes in different sites of CRC. The GO, BP and KEGG pathway enrichment analyses were performed for differentially expressed genes in different sites of CRC. The blue module, red module, turquoise module, and yellow module at the horizontal axis represent the transverse colon cancer, rectum cancer, sigmoid cancer, and ascending colon cancer, respectively. The vertical axis represents the GO, BP pathway and the KEGG pathway. The beginning of hsa and GO represents the KEGG pathway and GO, BP, respectively. The bubble size represents the number of genes enriched, and the colour ranges from blue to red represents the size of the p-value. [file 12967_2019_2102_MOESM4_ESM.tif]
